# Chemopreventive effect of Betulinic acid via mTOR -Caspases/Bcl2/Bax apoptotic signaling in pancreatic cancer

**DOI:** 10.1186/s12906-020-02976-7

**Published:** 2020-06-08

**Authors:** Yangyang Guo, Hengyue Zhu, Min Weng, Cheng Wang, Linxiao Sun

**Affiliations:** Key Laboratory of Diagnosis and Treatment of Severe Hepato-Pancreatic Diseases of Zhejiang Province, Zhejiang Provincial Top Key Discipline in Surgery, Wenzhou Medical University First Affiliated Hospital, Wenzhou, Zhejiang China

**Keywords:** Betulinic acid, mTOR signaling, Apoptosis, Pancreatic cancer

## Abstract

**Background:**

Pancreatic cancer is aggressive with no symptoms until the advanced stage reached. The increased resistance of pancreatic cancer to chemotherapy demonstrates a dilemma in the clinical field. Hence, it is a matter of great urgency to develop an effective drug to treat patients with pancreatic cancer**.** Betulinic acid is a major triterpene isolated from spina date seed. Several studies have suggested its low toxicity and side effects to patients with malaria and inflammation. However, relevant studies on betulinic acid in inhibiting cancer were insufficient and the molecular mechanism was unclear. This study aimed to systematically explore the potential anti-cancer functions of betulinic acid in pancreatic cancer, and investigate its underlying molecular mechanism.

**Methods:**

The Counting Kit-8 assay, colony formation, transwell invasion assay, wound healing assay, flow cytometry and xenograft nude mice model were used to evaluate the effect of betulinic acid on the proliferation, invasion and migration ability of pancreatic cancer cells.

**Results:**

Our results showed that betulinic acid obviously suppressed pancreatic cancer both in vitro and in vivo in a dose-dependent manner. We also determined that betulinic acid inhibited pancreatic cancer by specifically targeting mTOR signaling rather than Nrf2 or JAK2.

**Conclusions:**

These findings clarify that betulinic acid is a potential and valuable anticancer agent for pancreatic cancer, and indicate the specific molecular target of betulinic acid.

## Background

Pancreatic cancer is one of the fatal malignancy in the world. Global Cancer Observatory (GCO, http://gco.iarc.fr) shows that approximate 400,000 people died from pancreatic cancer each year, ranking the seventh leading causes of cancer death [1]. The overall five-year survival rate of pancreatic cancer is far below 10% and the lowest of almost all types of cancers [2]. Surgery is considered to be the only potential treatment, followed by adjuvant chemotherapy. However, pancreatic cancer is not sensitive to most of the current chemotherapeutic drugs [3]. Over 80% of patients with pancreatic cancer are diagnosed when the lesion is not suitable for operation [2]. Therefore, it is urgent to develop an effective drug with less toxic and side effects to treat patients with pancreatic cancer.

Spina date seed has served as an anti-insomnia food therapy in Chinese history. Betulinic acid, a major natural product extracted from spina date seed, exhibits multiple biological activities such as anti-malarial, anti-inflammatory and anti-HIV [4]. Steele et al. have also suggested that betulinic acid can be an anti-malarial natural product both in vitro and in vivo experiments [5]. Jinbo et al. have demonstrated that betulinic acid can regulate the expression of inflammatory cytokines to improve inflammation [6]. In addition, betulinic acid can also interfere with HIV-1 maturation and inhibit its fusion [7]. The broad biological activities of betulinic acid against different types of cancer have been reported recently. However, the potential molecular mechanism and the specific intracellular targets of betulinic acid are unclear. The purpose of this study was to investigate the effects of betulinic acid on the pancreatic cancer cells, and to explore the molecular mechanism of betulinic acid. This study will provide a new idea for the diagnosis and treatment of pancreatic cancer, and further to deeply understand the anticancer mechanism of betulinic acid.

## Methods

### Drugs and antibodies

Betulinic acid was purchased from YuanYe biotechnology (Shanghai, USA) and dissolved in DMSO as 100 mM. Counting Kit-8 (CCK-8) assay and Annexin V-FITC Apoptosis kit were obtained from BestBio Company (Shanghai, China). mTOR antibody (ab2732), Caspase-3 antibody (ab2302), p62 antibody (ab155686) were provided by Abcam. After that, S6K1 antibody (CST 9202), p-S6K1 antibody (CST 9204S), AMPK antibody (CST 2532S), p-AMPKα1 antibody (CST 2537), p-mTOR antibody (CST 5536S), Caspase8 antibody (CST 4790), Bax antibody (CST 5023S) and LC3A/B antibody (CST 12741) were bought from Cell Signaling Technology. Bcl2 antibody (12789–1-AP) and GAPDH antibody (AP0063) were acquired from Proteintech and Bioworld Technology, respectively.

### Cells and cell culture

The American Type Culture Collection (ATCC, Manassas, VA, USA) provided human pancreatic cancer cell line PANC-1 and SW1990. Dulbecco’s modified Eagle’s medium (DMEM; GENOM, Hangzhou, China) supplemented with 10% fetal bovine serum (FBS; Thermo Fisher Scientific, Waltham, MA, USA) and 1% Penicillin-Streptomycin (Gibco/Thermo Fisher Scientific) were used to maintain the cells at 37 °C in a 5% CO_2_ humidified atmosphere. Cells were sub-cultured every 2–3 days.

### Cell viability assay

The proliferation of PANC-1 and SW1990 cells was measured by using CCK-8 assay according to the manufacturer’s instructions [8]. Cells were cultured in 96-well plates (5 × 10^3^/well) for 24 h and treated with the indicated concentrations of betulinic acid. Then, the cells were treated with 100 μl of CCK-8 solution and incubated in the dark for another 2 h at 37 °C. The viability of cells was quantified using a Multiskan Spectrum spectrophotometer (Thermo Fisher Scientific, Inc.) with the optical density (OD) at 450 nm. The following formula was used to calculate % cell viability:

$$ \%\mathrm{cell}\ \mathrm{viability}=\frac{{\mathrm{OD}}_{450}\left(\mathrm{treatedcells}\right)-{\mathrm{OD}}_{450}\left(\mathrm{blank}\ \mathrm{cells}\right)}{{\mathrm{OD}}_{450}\left(\mathrm{controlcells}\right)-{\mathrm{OD}}_{450}\left(\mathrm{blank}\ \mathrm{cells}\right)}\times 100 $$

### Real time cellular analysis

The proliferation assay was measured via cell culture E16-Plate (ACEA Biosciences, San Diego, USA) at 2 × 10^5^ cells/well. Label-free Real-time Cellular Analysis System (RTCA; Roche, Penzberg, Germany) was applied to automatically record the cell growth index and normalized at every time point following treatment.

### Colony formation assay

The cells were plated in 6-well plates at a density of 500–1000 cells/well. The cells were treated with betulinic acid after the cells growth can be visible to the naked eye. After 24 h of treatment, cell colonies were fixed with formaldehyde and stained with crystal violet for counting [9].

### Immunofluorescence

PANC-1 and SW1990 human pancreatic cancer cells were grown on glass coverslips at a density of 5 × 10^3^ cells/well, and fixed in 4% formaldehyde for 15 min. Then cells were permeabilized with 0.1% Triton X-100, and blocked in 4% normal goat serum in PBS for 1 h. Immunofluorescence staining was performed using primary antibodies against Ki67 (1:100; Cell Signaling). Appropriate secondary antibodies were obtained from Santa Cruz.

### Migration assay

The exponentially growing cells were seeded in 6-well plates at a density of 5× 10^5^ cells/well and incubated at 37 °C for 48 h. After that, a crystal pipette tip was used to scratch the culture area to create a linear gap in the confluent cell monolayer. Detached cells were washed away with PBS, followed by adding betulinic acid to fill the linear gap. An inverted microscope was then adopted to capture images of the culture area every 24 h [10].

### Transwell assay

The invasion capacity of PANC-1 and SW1990 cells in vitro was assessed by Transwell (Costar, New York, NY, USA) assay. Cells cultured on 500 μl of serum-free medium at density of 1 × 10^5^ cells with betulinic acid were inoculated in the upper chamber, followed by plastered with reduced growth factor Matrigel® to do the invasion assay. Meanwhile, a medium containing 10% FBS was added into the lower chamber as a chemoattractant. After incubating for a while, Q-tip was used to remove cells lying on the upper surface of the membrane. Formaldehyde and 0.5% crystal violet (Sigma) were then used to fix and stain the invaded cells in correct order. To ensure the accuracy of the counting, five random fields under a microscope were selected to calculate the number of invaded cells [9].

### Flow cytometry analysis

The cells were treated with betulinic acid in a 6-well plate (5 × 10^5^/ml, 2 ml/well), and washed with PBS. The cells were harvested and resuspended in binding buffer at a density of 5 × 10^5^ cells/ml when the cells reached 85% confluence. Annexin V-FITC (5 μl) was used to incubate cells at room temperature for 15 min, then added 5 μl propidium iodide (PI) for another 5 min. All the incubation processes were performed under dark reaction conditions. In the end, flow-cytometry was carried out using a FACS C6 instrument, and data were analyzed using FlowJo 7.6 (USA).

### Protein extraction and western blotting

Following treatment with different concentrations of betulinic acid, the cells were lysed in ice-cold RIPA lysis buffer (Beyotime, Shanghai, China) supplemented with 10% PhosSTOP (Roche, Basel, Switzerland), 1% PMSF (Beyotime, Shanghai, China) and 1% DTT. After incubation on ice for 30 min, the cells were collected by centrifugation for 10 min (12,000×g, 4 °C). The protein concentration was calculated using a Pierce BCA protein assay kit (Beyotime, Shanghai, China) from the supernatant. Total protein was subjected to 12% SDS-PAGE before transferred onto PVDF membranes (Bio-Rad Laboratories, Inc.). Membranes were blocked with 5% skim non-fat milk in TBS-T for 1 h at room temperature, followed by incubating with antibodies at 4 °C overnight. After three 7–10 min washing in TBST, membranes were incubated with the secondary antibodies for another 1 h at room temperature. Another three 5-min washing were done with TBST, and the protein bands were visualized using chemiluminescence detection on autoradiographic film. Image-Pro Plus was used to detect the intensity of signals for quantification. Normalization was required according to GAPDH antibody.

### Nude mouse tumorigenicity assay

Male nude mice (BALB/c) were obtained from the Experimental Animal Centre of Wenzhou Medical University (Wenzhou, China). The inclusion criteria were mice aged 6–8 wks old and weighing 18–22 g. Mice were fed standard chow and water in the environment with controlled temperature, humidity and light, followed by a night of fasting the day before the experiment. 5 × 10^6^ PANC-1 cells in 100 μl of PBS were injected subcutaneously in the left neck of experimental mice (*n* = 5), then intragastric administration of betulinic acid (40 mg/kg·d) for 30 days. In the meantime, another 5 model mice received injection of 5 × 10^6^ PANC-1 cells (control group). The size of tumors were monitored daily until they become bulky or necrotic. The formula: V = (length×width^2^)/2 was defined to assess the tumor volume, length was always the longest dimension [11]. After the monitoring, these mice were killed by a lethal dose of carbon dioxide to examine tumor formation. The animal experiments including animals’ euthanasia were performed in compliance with all regulatory institutional guidelines for animal welfare (National Institutes of Health Publications, NIH Publications No. 80–23). The guidelines were approved by the Institutional Review Board of Wenzhou Key Laboratory of Surgery, and the Institutional Animal Care and Use Committee of Wenzhou Medical University, China.

### Histopathological examination

After fixing in formalin, the tumor specimens were embedded in paraffin and then cut into 4-μm sections. Hematoxylin and eosin (HE, Yuanye Biotechnology, Shanghai, China) were used to stain the sections. DM4000 B LED microscope system (Leica Microsystems, Germany) and DFC420C 5 M digital microscope camera (Leica Microsystems) were applied to examine and take photos of slides, respectively.

### Statistical analysis

SPSS 18.0 (IBM, Armonk, USA) and GraphPad Prism 6.0 (GraphPad Software Inc., San Diego, CA, USA) were used for statistical analysis (mean ± standard deviation). The mean of each pair groups was compared by One-way ANOVA and the Student-Newman Keuls tests. *P* < 0.05 was considered as statistically significant. LSD method was then used for intergroup comparison if the analysis of variance were statistically significant.

## Results

### Cell proliferation inhibitory effect of betulinic acid on pancreatic cancer

**Fig. 1:**
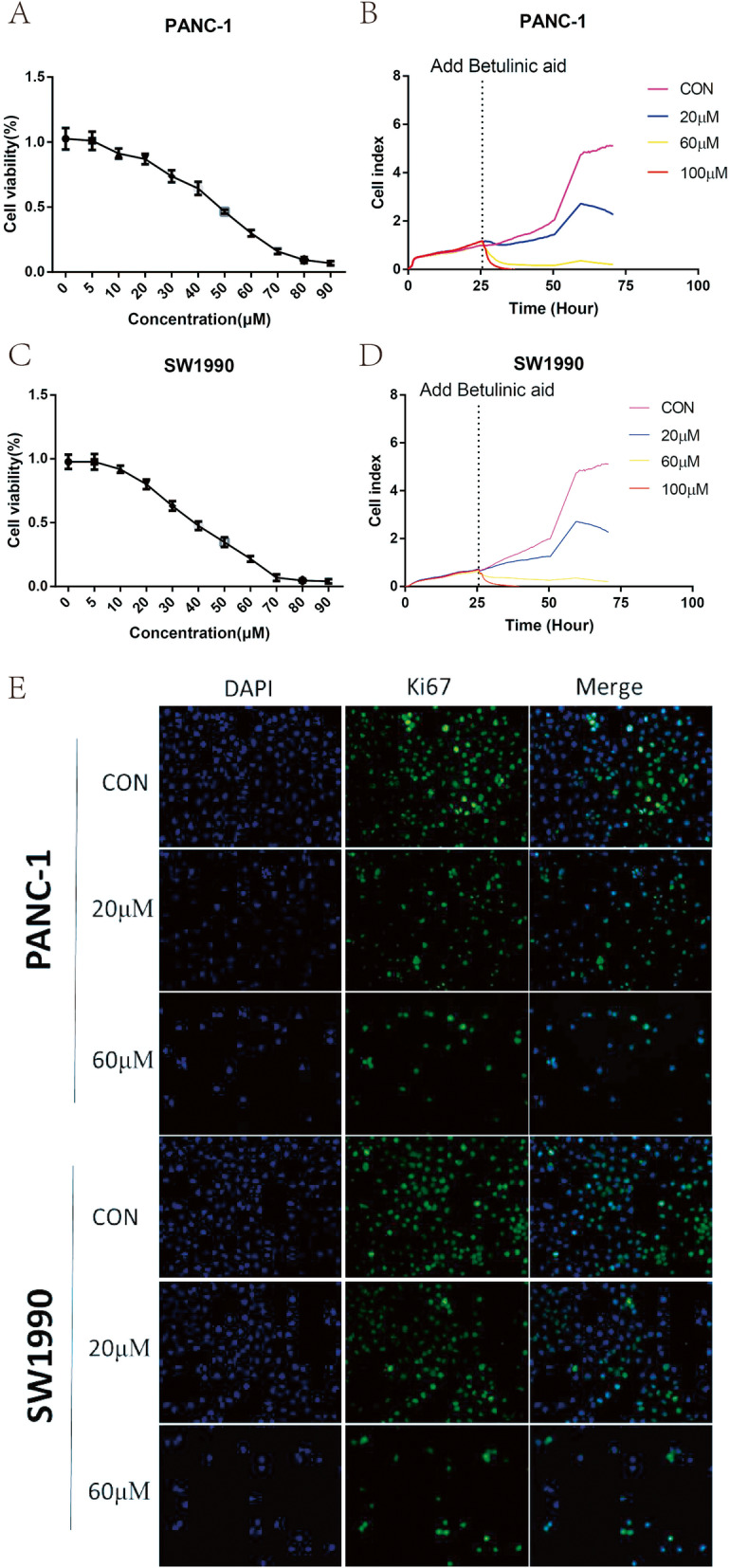
Betulinic acid inhibits PANC-1 and SW1990 cells proliferation. CCK8 assay of PANC-1(**a**) and SW1990 (**c**) cells incubated with 5 μM,10 μM, 20 μM, 30 μM, 40 μM, 50 μM, 60 μM, 70 μM, 80 μM, 90 μM. Betulinic acid or an equal volume of DMEM medium for 24 h. Label-free Real-time Cellular Analysis (RTCA) following PANC-1 (**b**) and SW1990 (**d**) cells incubated with betulinic acid (20 μM, 60 μM) or an equal volume of DMEM medium for 24 h. (**e**) Ki67 Immunofluorescence following PANC-1 and SW1990 cells incubated with betulinic acid (20 μM, 60 μM) or an equal volume of DMEM medium for 24 h

**Fig. 2:**
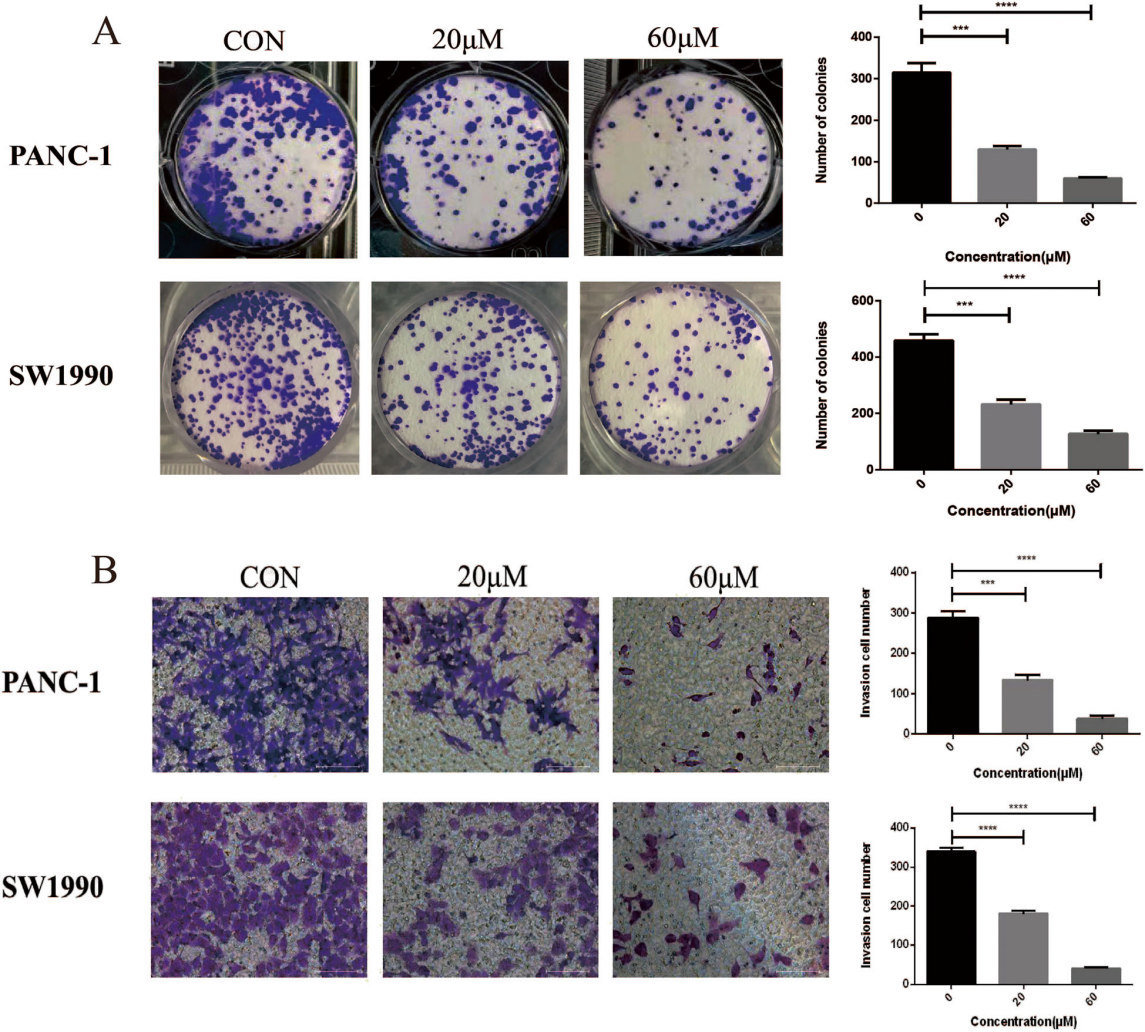
Betulinic acid inhibit PANC-1 and SW1990 cells Colony formation and invasion. **a** Colony formation assay of PANC-1 and SW1990 cells incubated with betulinic acid (20 μM, 60 μM) or an equal volume of DMEM medium for 24 h. *Data are presented as mean ± SD, *N* = 3; *, *P* < 0.05;**, *P* < 0.01; ***, *P* < 0.001; ****, *P* < 0.0001, compared with control. **b** Transwell assay following PANC-1 and SW1990 cells incubated with Betulinic acid (20 μM, 60 μM) or an equal volume of DMEM medium for 24 h. *Data are presented as mean ± SD, *N* = 3; *, *P* < 0.05; **, *P* < 0.01; ***, *P* < 0.001; ****, *P* < 0.0001, compared with control

CCK-8 assay, RTCA, colony formation assay and Ki67 Immunofluorescence were conducted to detect the antitumor effect of betulinic acid on the proliferation of PANC-1 and SW1990 cells. As shown in Fig. 1a and c, the rate of cell viability in betulinic acid-treated cells was significantly decreased compared with control cells (*P* < 0.05). The half maximal inhibitory concentration values of betulinic acid for PANC-1 and SW1990 cells at 24 h were 47 and 38 μM, respectively. The antitumor effect of betulinic acid on both PANC-1 and SW1990 cells were subsequently monitored (Fig. 1b and d). RTCA showed that the cell proliferation of PANC-1 and SW1990 reduced remarkably after treatment with 20 and 60 μM betulinic acid than the DMSO. Plate colony formation assays were performed to detect the proliferation of PANC-1 and SW1990 cells after treatment with betulinic acid (Fig. 2a), which revealed that betulinic acid potently inhibited the proliferation and colony formation of PANC-1 and SW1990 cells. These results indicated that Betulinic acid treatment inhibited the proliferation of PANC-1 and SW1990 cells in a dose-dependent manner.

### The inhibition of betulinic acid on invasion and migration

**Fig. 3:**
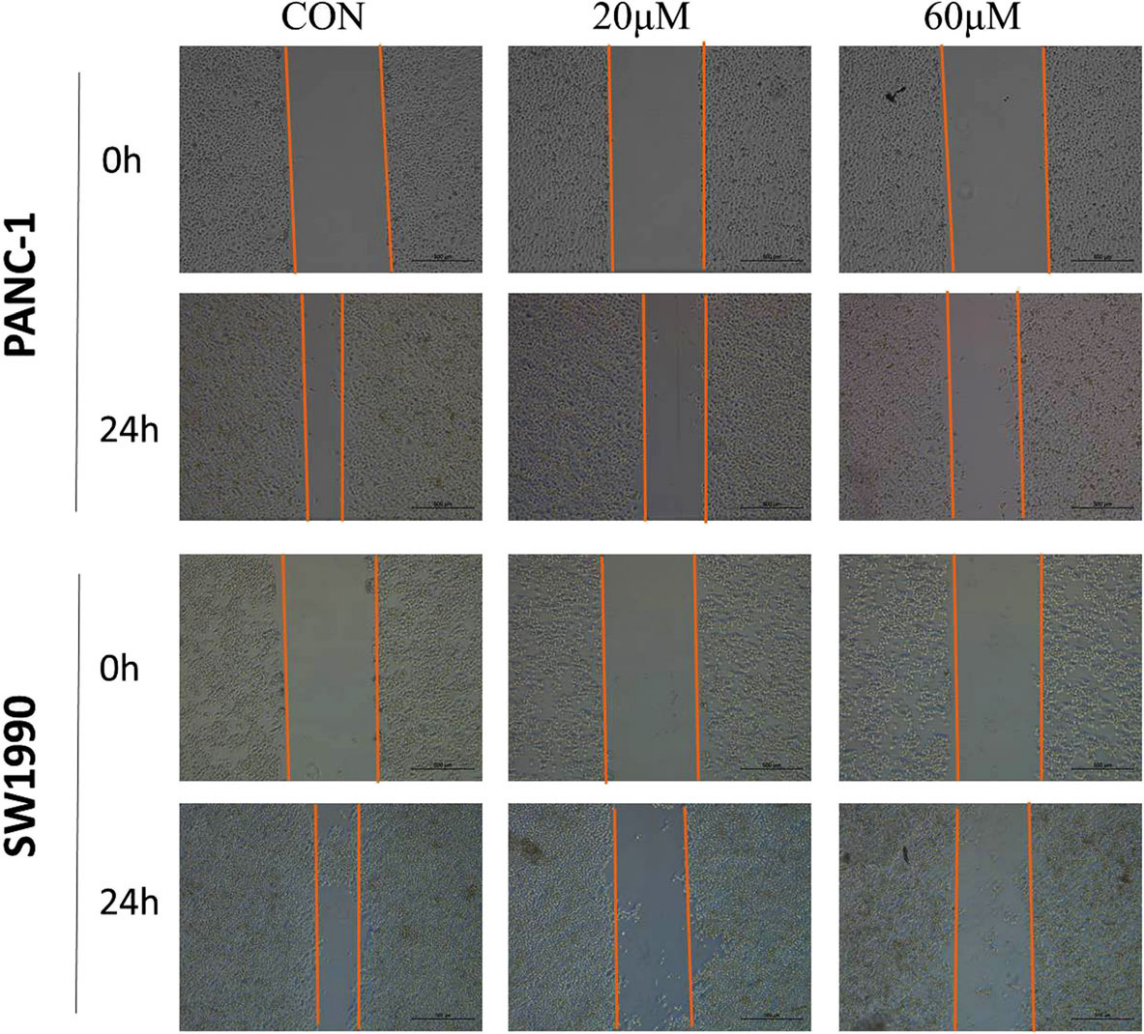
Betulinic acid inhibits PANC-1 and SW1990 cells migration. Wound healing assay following PANC-1 and SW1990 cells incubated with betulinic acid (20 μM, 60 μM) or an equal volume of DMEM medium for 24 h

In addition, we also carried out cell invasion by Transwell test, and determined the ability of cells migration with betulinic acid by wound healing experiment. Transwell assay showed that PANC-1 and SW1990 cells with DMSO had strong invasive ability (Fig. 2b). Compared with the vehicle group, cells treated with betulinic acid inhibited cell invasion significantly. Consistently, the migration ability of cells decreased gradually after adding 20 and 60 μM betulinic acid. Wound healing experiments showed that betulinic acid remarkably suppressed the migration of PANC-1 cells and SW1990 cells (Fig. 3). These results suggests that betulinic acid inhibits the invasion and migration of Panc-1 and SW1990 cells in a dose-dependent manner.

### Betulinic acid induces pancreatic cancer cells apoptosis

**Fig. 4:**
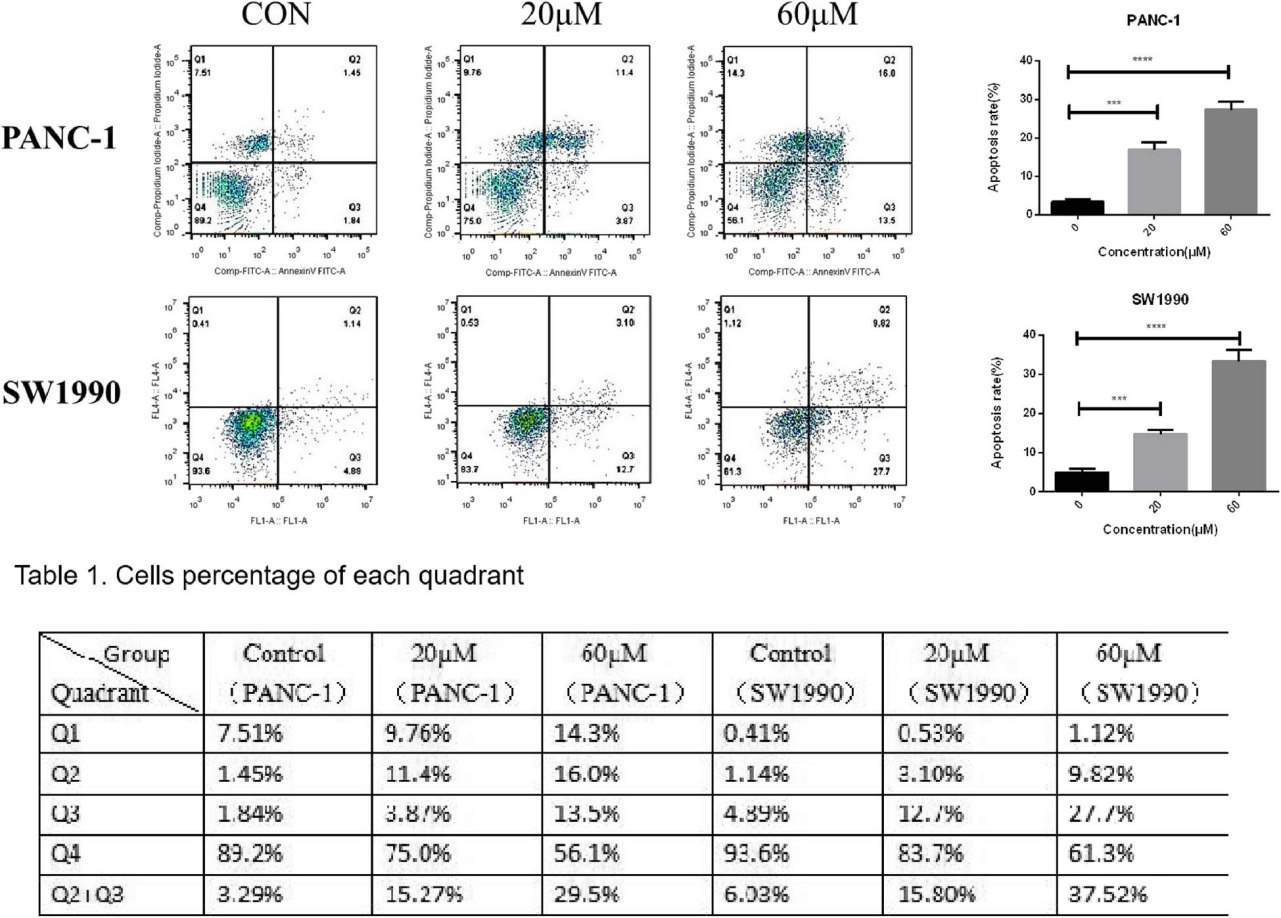
Betulinic acid promotes PANC-1 and SW1990 cells apoptosis. Flow cytometry for apoptosis [apoptosis ratio was calculated as (Q2 + Q3)/(Q1 + Q2 + Q3 + Q4)] of PANC-1 and SW1990 cells incubated with Betulinic acid (20 μM, 60 μM) or an equal volume of DMEM medium for 24 h.. *Data are presented as mean ± SD, N = 3; **, *P* < 0.01; ***, *P* < 0.001; ****, *P* < 0.0001, compared with control

To investigate the effect of betulinic acid on apoptosis of pancreatic cancer cell lines, PANC-1 and SW1990 cells were treated with betulinic acid at concentrations of 0, 20 and 60 μM for 24 h. Apoptosis was identified by AnnexinV-FITC/PI method, which showed that the percentage of apoptosis of PANC-1 cells treated with betulinic acid increased from 3.29 to 29.5% and from 6.03 to 37.52% in SW1990 cells (Fig. 4). With the increase of betulinic acid concentration, the degree of apoptosis of PANC-1 and SW1990 cells enhanced. These findings shows that betulinic acid treatment does dependently promotes apoptosis of PANC-1 and SW1990 cells.

### Betulinic acid inducing apoptosis depends on mTOR signaling

**Fig. 5:**
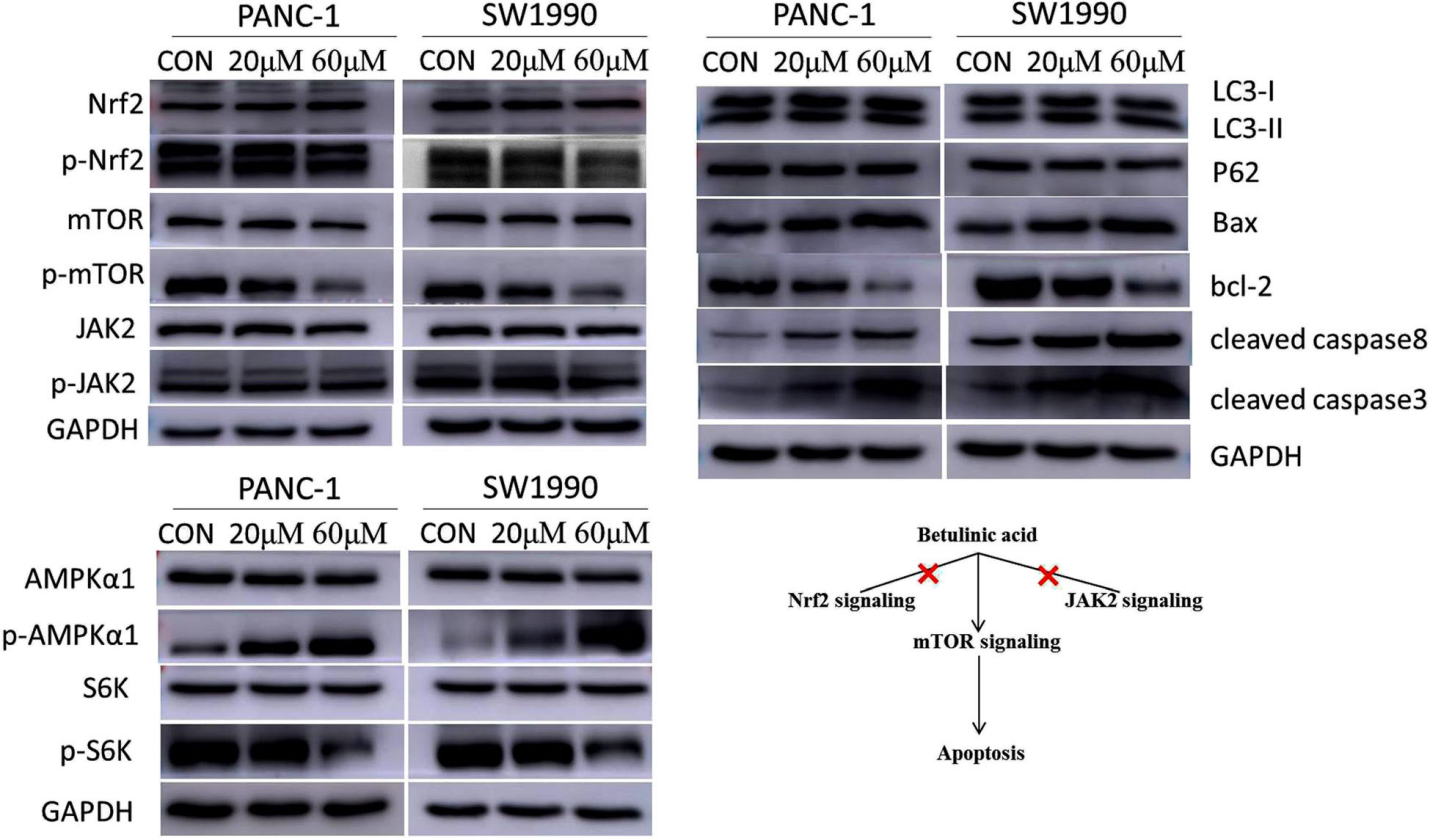
Betulinic acid inducing apoptosis is dependent on mTOR/S6K1-Caspases/Bcl2/Bax apoptotic signaling. After 24 h treatment with Betulinic acid (20 μM, 60 μM), the expression of AMPK/mTOR signaling pathway autophagy and apoptosis-related proteins was detected by western blotting analysis

We further determine the possible mechanism of apoptosis induced by betulinic acid, the expression of apoptosis-related and autophagy-related proteins was detected using western blotting. As showed in Fig. 5, betulinic acid treatment increased the expression of cleaved caspase3, cleaved caspase8 and Bax, while the anti-apoptotic protein Bcl-2 was down-regulated, which further confirmed the apoptosis induction of PANC-1 and SW1990 cells by betulinic acid. We also found that there was no significant change in LC-3B and p62 in betulinic acid treatment group, indicating that betulinic acid had no effect on autophagy of PANC-1 and SW1990 cells. In addition, in PANC-1 and SW1990 cells, p-mTOR was down-regulated, while p-AMPK was up-regulated, showing that AMPK/mTOR signal transduction was involved in autophagy and apoptosis induced by betulinic acid. Furthermore, the treatment of betulinic acid down-regulated the expression of p-S6K in PANC-1 and SW1990 cells, suggesting that protein synthesis was also inhibited. We speculated that betulinic acid may inhibit the proliferation and apoptosis of pancreatic cancer cells by promoting the activation of AMPK, inhibiting the activation of mTOR, as well as inducing autophagy and inhibiting protein synthesis.

### Betulinic acid inhibits tumor growth in xenograft nude mice model

**Fig. 6:**
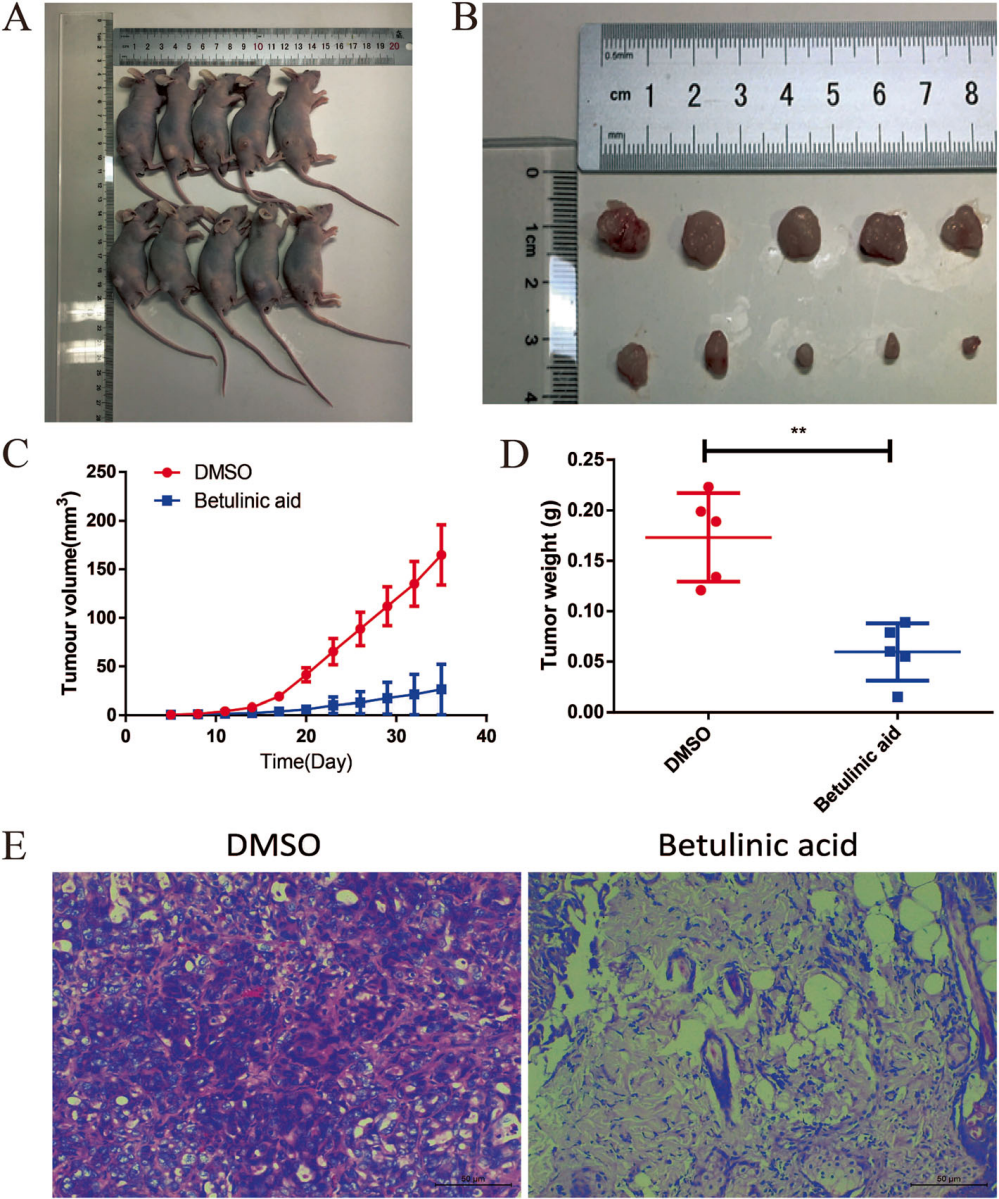
Betulinic acid inhibits tumor growth of cell xenografts in nude mice. To further verify the effect of the Betulinic acid on PDAC cells, PANC-1 cells xenograft tumors were treated with Betulinic acid. When the diameter of the tumors reached 1 mm, the mice were randomly divided to two groups with five mice in each group. After 30 days of treatment, the mice were killed (**a**) and the tumors were exfoliated (**b**). The tumor volume (**c**) was measured every three day for 30 days. Tumors weight (**d**) was measured after tumors exfoliated. **e** HE stain showed that Betulinic acid significantly inhibits tumor growth of cell xenografts in nude mice. One-way ANOVA with Tukey’s multiple comparison tests was utilized to analyze the subcutaneous tumor growth. All the experiments were performed in triplicate and the data are presented as the mean ± SD. The t-test was used for data analysis. *P < 0.05, **P < 0.01

From Fig. 6a and b, a significant difference in tumor volume was found from the tumor image after 30 days. The mean value of tumor volume and weight showed statistically significant differences between groups (Fig. 6c and d). After treatment with betulinic acid 40 mg/kg, the tumor volume and weight of transplanted PANC-1 cells decreased dramatically. In addition, a small number of tumor cells were observed in the betulinic acid treatment group, while a lot of tumor cells were observed in the control group (Fig. 6e). These results reveals that betulinic acid inhibits tumor growth in vivo.

## Discussion

Pancreatic cancer is one of the fatal malignancy in both sexes around the world. Several first-line chemotherapy agents such as gemcitabine have successfully improved survival of patients with different cancers, however, these agents met with limited outcomes in pancreatic cancer. The effective drug for treating pancreatic cancer is still restrained [12, 13]. Recent studies have showed several natural products may be novel candidates for developing pancreatic cancer therapeutics [3]. Such as Bangladeshi medicinal plant extracts, which showed obvious cytotoxicity to three pancreatic cancer cell lines with low toxicity [14], while Betulinic acid is a triterpene mainly derived from spina date seed. In China, spina date seed is widely used for insomnia therapy [15]. Yogeeswari et al. have suggested a variety of biological activities of betulinic acid include anti-inflammatory sterilization, human immunodeficiency virus (HIV) suppression and cytotoxicity against several tumors [16]. However, studies on the effect of betulinic acid in pancreatic cancer is insufficient, and the potential molecular mechanism of anti-tumor activities of betulinic acid remains unknown. In this study, we showed the inhibition of betulinic acid on cell proliferation, invasion and migration of two pancreatic cancer cell lines. Moreover, we engaged the xenograft nude mice model to confirm the anti-tumor bioactivity of betulinic acid in vivo.

The abnormal proliferation, invasion and migration ability of cancer cells require characteristic alterations on several pivotal signaling pathways. Nuclear factor erythroid 2-related factor 2 (Nrf2) signaling pathway can not only response to cellular stress, but also activate Nrf2-dependent transcriptional programs to further promote cancer hallmark proteins [17]. Cancer cells via facilitating Nrf2 signaling to circumvent the inhibition of autophagy, which means the activation of Nrf2 signaling acts as a protection of cancer cells [18]. Janus kinase 2 (JAk2) signaling pathway has participated in regulating immune system and cell growth [19]. JAK2 has been widely known for its high mutation in myeloproliferative neoplasms (~ 96% cancer patients with V617 mutation in exon 14 of JAK2) [20]. This leads to the development of several effective JAK2 inhibitors in clinical therapy, such as ruxolitinib [21]. However, betulinic acid in our study had no effects on Nrf2 and JAK2, but significant inhibited the phosphorylation of mTOR in a dose-dependent manner in two pancreatic cancer cell lines (PANC-1 and SW1990) (Fig. 5). This indicates that the inhibition of pancreatic cancer cells by betulinic acid is through the targeting of mTOR signaling, rather than Nrf2 or JAK2.

The mammalian target of rapamycin (mTOR) signaling pathway is crucial in cell growth and division via regulating autophagy, apoptosis and other critical intracellular processes [22]. Studies based on prevailing cancer models have indicated that the abnormal activation of mTOR signaling drives tumorigenesis in a p53 independent manner [23]. Owing to the important role of mTOR signaling in oncogenesis, mTOR inhibitors such as rapamycin have great expectations in clinical chemotherapy, but their toxicity and side effects on normal cells are difficult to predict [24]. Recent novel natural products such as curcumin with hypo-toxicity targeting mTOR have showed a new therapeutic method via inhibiting mTOR signaling pathway [25]. Our results also clarified that betulinic acid was a specific mTOR inhibitor (Fig. 5). Furthermore, the expression changes of phosphorylated AMPKα1 (upstream inhibiting protein of mTOR) and S6K1 (direct downstream substrate of mTOR) confirmed the specificity of betulinic acid on mTOR. These results collectively demonstrated that betulinic acid might be a potential and valuable mTOR inhibitor with hypo-toxicity in pancreatic cancer.

As a valuable cancer therapy targets, mTOR signaling pathway has mainly modulated cancer cell autophagy or induced apoptosis [26, 27]. The nutritional status, growth factor and other environmental stresses caused by mTORC1 complex can inhibit autophagy process to affect cancer cells [28]. Recently, the mTOR signaling pathway has also been reported to induce the apoptosis of non-small lung cancer [26], esophageal cancer [29] and myeloid leukemia cancer [30]. Nevertheless, the exact molecular mechanism of mTOR signaling regulated by betulinic acid in pancreatic cancer remains unclear. Our data showed that autophagy markers like LC3-I, LC3-II and P62 were not affected by betulinic acid, whereas apoptosis related proteins including Bax, bcl-2, cleaved caspase 8 and cleaved caspase 3 were regulated by betulinic acid in a dose-dependent manner (Fig. 5). Based on all above data, we speculated that betulinic acid may specifically inhibit mTOR signaling via inducing apoptosis in pancreatic cancer. Consequently, our study demonstrated that betulinic acid inhibited pancreatic cancer both in vitro and in vivo. The inhibition effect of betulinic acid may through targeting mTOR signaling to specifically active Caspases/Bcl2/Bax apoptotic signaling. Further studies are required to explore the bioactive structure of betulinic acid and responsive domain of mTOR.

## Conclusion

Based on a variety of cell and mouse experiments, our results showed that betulinic acid can obviously suppress pancreatic cancer both in vitro and in vivo in a dose-dependent manner, which expands the anticancer class of betulinic acid. Furthermore, we explored the potential mechanism by which betulinic acid inhibited pancreatic cancer, and found that betulinic acid induces apoptosis by specifically through targeting mTOR signaling rather than Nrf2 or JAK2. These findings point out that betulinic acid acts as a potential and valuable anticancer agent for pancreatic cancer and indicate the specific molecular target of betulinic acid.

## Supplementary information

**Additional file 1.**

## Data Availability

The datasets used and analyzed during this study would be available upon request from the corresponding author.

## Abbreviations

GCO: Global Cancer Observatory
mTOR: Mammalian target of rapamycin
Nrf2: Nuclear factor erythroid 2-related factor 2
JAK2: Janus kinase 2
S6K1: Ribosome protein subunit 6 kinase 1
AMPK: AMP-activated protein kinase
HIV: Human immunodeficiency virus
CCK-8: Counting Kit-8
PBS: Phosphate buffer saline
FBS: Fetal bovine serum
