# Correction to: Chemopreventive effect of Betulinic acid via mTOR -Caspases/Bcl2/Bax apoptotic signaling in pancreatic cancer

**DOI:** 10.1186/s12906-021-03254-w

**Published:** 2021-04-26

**Authors:** Yangyang Guo, Hengyue Zhu, Min Weng, Cheng Wang, Linxiao Sun

**Affiliations:** Key Laboratory of Diagnosis and Treatment of Severe Hepato-Pancreatic Diseases of Zhejiang Province, Zhejiang Provincial Top Key Discipline in Surgery, Wenzhou Medical University First Affiliated Hospital, Wenzhou, Zhejiang China

**Correction to: BMC Complement Med Ther 20, 178 (2020)**

**https://doi.org/10.1186/s12906-020-02976-7**

Following publication of the original article [1], the authors reported that a mistake in Fig. 1b and d and  the group is not marked in Fig. 6b and d.

**Fig. 1:**
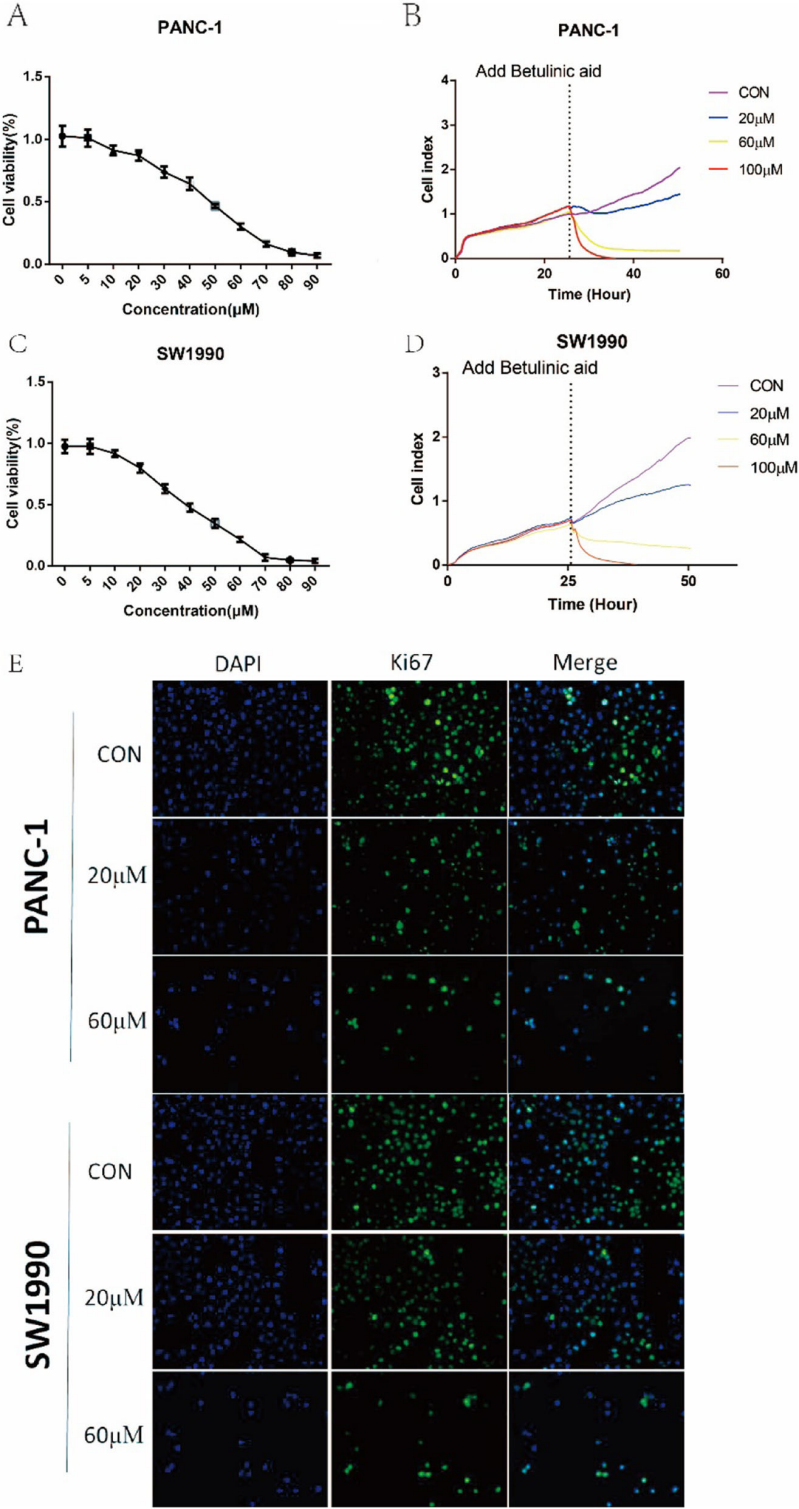
Betulinic acid inhibits PANC-1 and SW1990 cells proliferation. CCK8 assay of PANC-1 (**a**) and SW1990 (**c**) cells incubated with 5 μM,10 μM, 20 μM, 30 μM, 40 μM, 50 μM, 60 μM, 70 μM, 80 μM, 90 μM. Betulinic acid or an equal volume of DMEM medium for 24 h. Label-free Real-time Cellular Analysis (RTCA) following PANC-1 (**b**) and SW1990 (**d**) cells incubated with betulinic acid (20 μM, 60 μM) or an equal volume of DMEM medium for 24 h. (**e**) Ki67 Immunofluorescence following PANC-1 and SW1990 cells incubated with betulinic acid (20 μM, 60 μM) or an equal volume of DMEM medium for 24 h

**Fig. 6:**
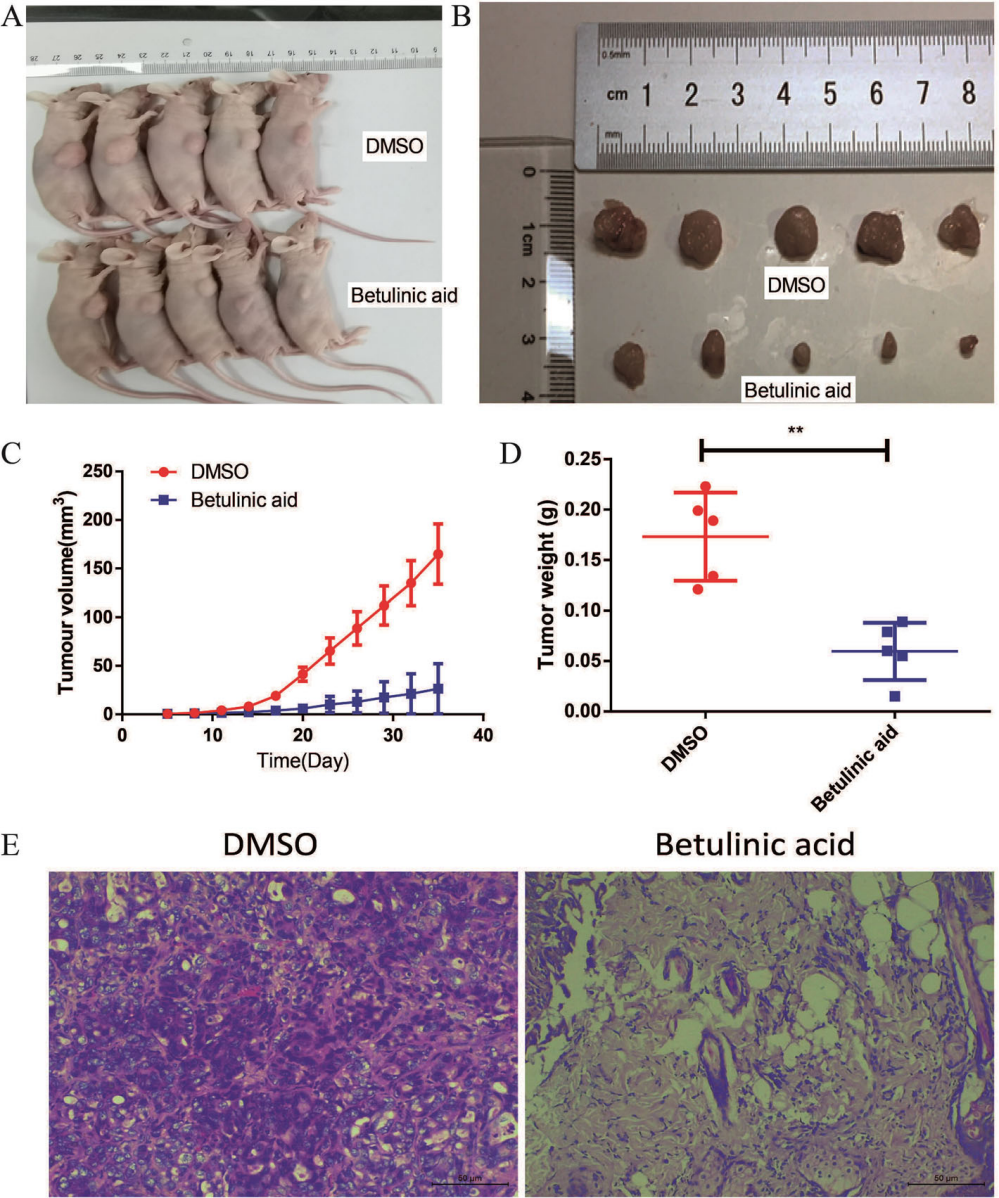
Betulinic acid inhibits tumor growth of cell xenografts in nude mice. To further verify the effect of the Betulinic acid on PDAC cells, PANC-1 cells xenograft tumors were treated with Betulinic acid. When the diameter of the tumors reached 1 mm, the mice were randomly divided to two groups with five mice in each group. After 30 days of treatment, the mice were killed (**a**) and the tumors were exfoliated (**b**). The tumor volume (**c**) was measured every three day for 30 days. Tumors weight (**d**) was measured after tumors exfoliated. **e** HE stain showed that Betulinic acid significantly inhibits tumor growth of cell xenografts in nude mice. One-way ANOVA with Tukey’s multiple comparison tests was utilized to analyze the subcutaneous tumor growth. All the experiments were performed in triplicate and the data are presented as the mean ± SD. The *t*-test was used for data analysis. *P < 0.05, **P < 0.01

The original article [1] has been updated.

## References

[1] Guo Y, Zhu H, Weng M (2020). Chemopreventive effect of Betulinic acid via mTOR -Caspases/Bcl2/Bax apoptotic signaling in pancreatic cancer. BMC Complement Med Ther.

