# Supplementary material for: Chemopreventive effect of Betulinic acid via mTOR -Caspases/Bcl2/Bax apoptotic signaling in pancreatic cancer
Source: BMC Complement Med Ther. 2020 Jun 8;20:178. doi: 10.1186/s12906-020-02976-7 (PMC7282238; doi:10.1186/s12906-020-02976-7)

Nrf2

kDa


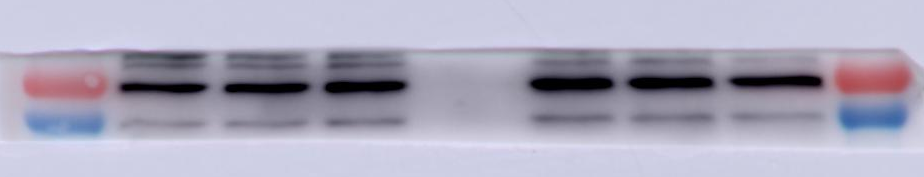


63

75

SW1990：CON 20μM 60μM

PANC-1：CON 20μM 60μM

p-Nrf2

kDa


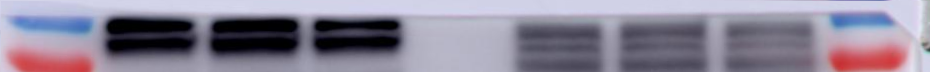


100

75

SW1990：CON 20μM 60μM

PANC-1：CON 20μM 60μM

mTOR

kDa


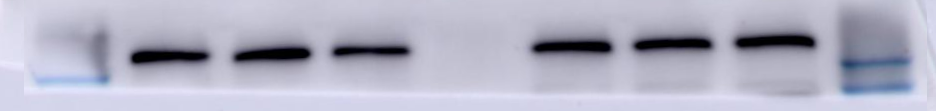


SW1990：CON 20μM 60μM

PANC-1：CON 20μM 60μM

245

p-mTOR

kDa


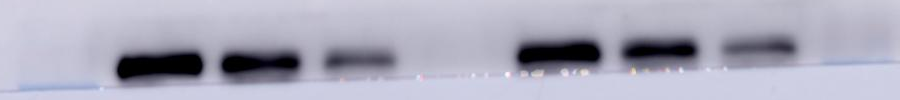


245

SW1990：CON 20μM 60μM

PANC-1：CON 20μM 60μM

JAK2

kDa


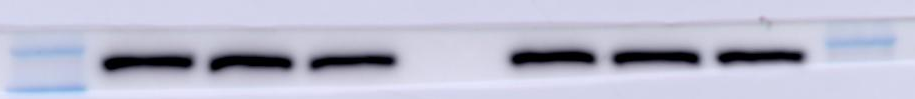


135

SW1990：CON 20μM 60μM

PANC-1：CON 20μM 60μM

p-JAK2

kDa

135


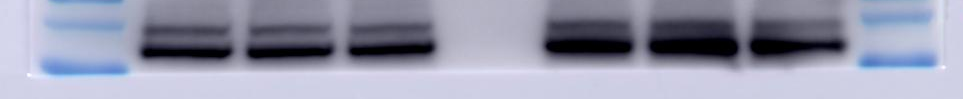


100

SW1990：CON 20μM 60μM

PANC-1：CON 20μM 60μM

GAPDH

kDa


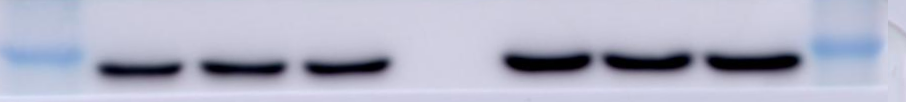


48

SW1990：CON 20μM 60μM

PANC-1：CON 20μM 60μM

LC3

kDa


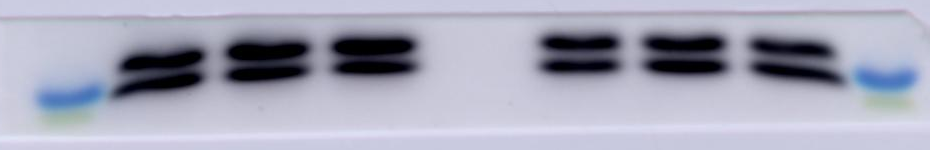


10

17

SW1990：CON 20μM 60μM

PANC-1：CON 20μM 60μM

P62

kDa


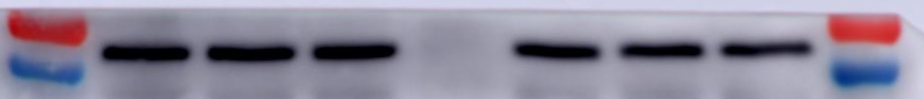


55

72

SW1990：CON 20μM 60μM

PANC-1：CON 20μM 60μM

Bax

kDa


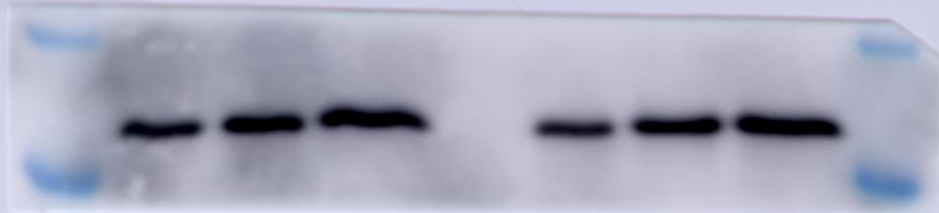


17

26

SW1990：CON 20μM 60μM

PANC-1：CON 20μM 60μM

bcl-2

kDa


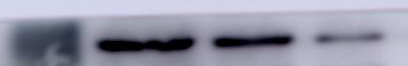


26

PANC-1：CON 20μM 60μM

bcl-2

kDa


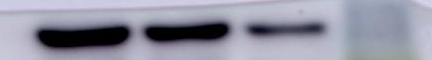


26

SW1990：CON 20μM 60μM

Cleaved caspase 8

kDa

43


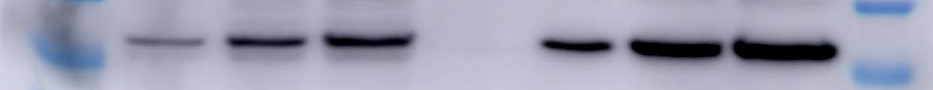


34

SW1990：CON 20μM 60μM

PANC-1：CON 20μM 60μM

Cleaved caspase 3

kDa


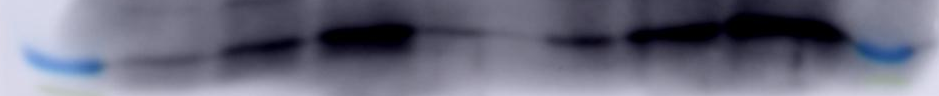


17

SW1990：CON 20μM 60μM

PANC-1：CON 20μM 60μM

GAPDH

kDa


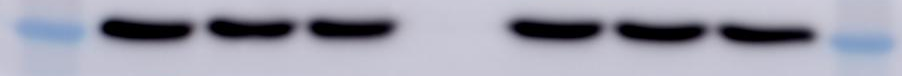


35

SW1990：CON 20μM 60μM

PANC-1：CON 20μM 60μM

AMPKα1

kDa


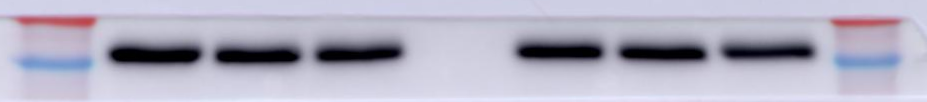


63

75

SW1990：CON 20μM 60μM

PANC-1：CON 20μM 60μM

p-AMPKα1

kDa


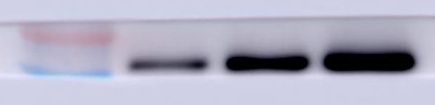


63

75

PANC-1：CON 20μM 60μM

p-AMPKα1

kDa


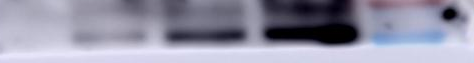


63

SW1990：CON 20μM 60μM

S6K

kDa

75


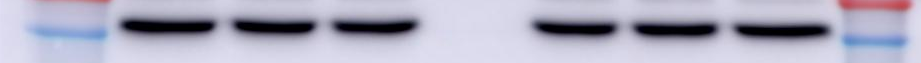


63

SW1990：CON 20μM 60μM

PANC-1：CON 20μM 60μM

p-SK6

kDa


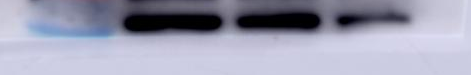


63

PANC-1：CON 20μM 60μM

p-SK6

kDa


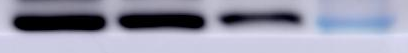


63

SW1990：CON 20μM 60μM

GAPDH


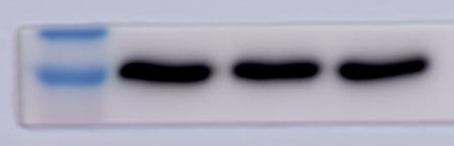


35

kDa

PANC-1：CON 20μM 60μM

GAPDH


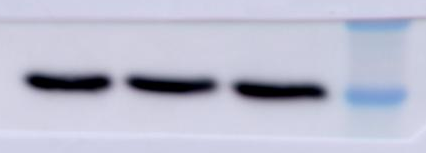


35

kDa

SW1990：CON 20μM 60μM


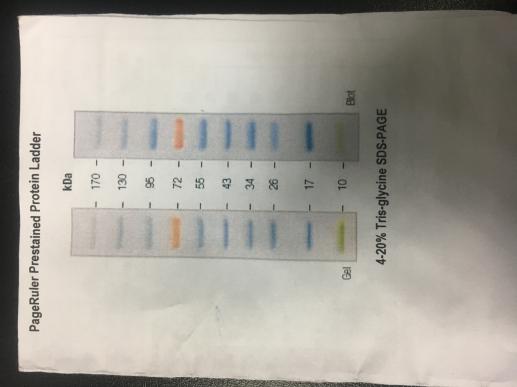

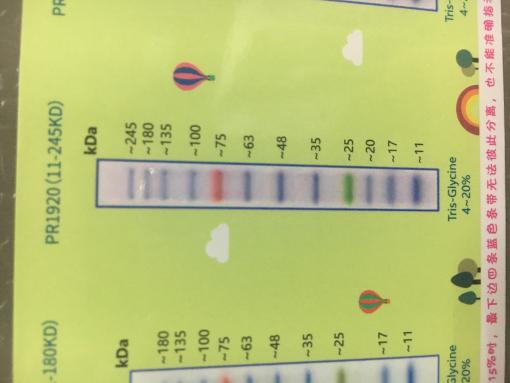

Supplement: Supplementary file 1 — Additional file 1. [file 12906_2020_2976_MOESM1_ESM.docx]
